# Distal Aortic Failure Following the Frozen Elephant Trunk Procedure for Aortic Dissection

**DOI:** 10.3389/fcvm.2022.911548

**Published:** 2022-06-06

**Authors:** Tim Berger, Miriam Graap, Bartosz Rylski, Albi Fagu, Roman Gottardi, Tim Walter, Philipp Discher, Muhammad Taha Hagar, Stoyan Kondov, Martin Czerny, Maximilian Kreibich

**Affiliations:** ^1^Department of Cardiovascular Surgery, Faculty of Medicine, University Hospital Freiburg Heart Centre, University of Freiburg, Freiburg, Germany; ^2^Department for Diagnostic and Interventional Radiology, Faculty of Medicine, Medical Centre-University of Freiburg, Albert-Ludwigs-University of Freiburg, Freiburg, Germany

**Keywords:** aortic dissection, frozen elephant trunk (FET), distal aortic failure, aortic reintervention, dSINE

## Abstract

**Background:**

Aim of this study was to report and to identify risk factors for distal aortic failure following aortic arch replacement via the frozen elephant trunk (FET) procedure.

**Methods:**

One hundred eighty-six consecutive patients underwent the FET procedure for acute and chronic aortic dissection. Our cohort was divided into patients with and without distal aortic failure. Distal aortic failure was defined as: (I) distal aortic reintervention, (II) aortic diameter dilatation to ≥ 6 cm or > 5 mm growth within 6 months, (III) development of a distal stent-graft-induced new entry (dSINE) and/or (IV) aortic-related death. Preoperative, intraoperative, postoperative and aortic morphological data were analyzed.

**Results:**

Distal aortic failure occurred in 88 (47.3%) patients. Forty-six (24.7%) required a distal reintervention, aortic diameter dilatation was observed in 9 (4.8%) patients, a dSINE occurred in 22 (11.8%) patients and 11 (6.4%) suffered an aortic-related death. We found no difference in the number of communications between true and false lumen (*p* = 0.25) but there were significantly more communications between Ishimaru zone 6–8 in the distal aortic failure group (*p* = 0.01). The volume of the thoracic descending aorta measured preoperatively and postoperatively within 36 months afterward was significantly larger in patients suffering distal aortic failure (*p* < 0.001; *p* = 0.011). Acute aortic dissection (SHR 2.111; *p* = 0.007), preoperative maximum descending aortic diameter (SHR 1.029; *p* = 0.018) and preoperative maximum aortic diameter at the level of the diaphragm (SHR 1.041; *p* = 0.012) were identified as risk factors for distal aortic failure.

**Conclusion:**

The incidence and risk of distal aortic failure following the FET procedure is high. Especially those patients with more acute and more extensive aortic dissections or larger preoperative descending aortic diameters carry a substantially higher risk of developing distal aortic failure. The prospective of the FET technique as a single-step treatment for aortic dissection seems low and follow-up in dedicated aortic centers is therefore paramount.

## Introduction

Total aortic arch replacement via the frozen elephant trunk (FET) technique has rapidly evolved over the last decade with broadened indications for several acute and chronic aortic pathologies (1–4). The FET procedure was initially almost exclusively carried out by experienced cardiovascular surgeons, but it has since become a highly standardized procedure safely performed by junior surgeons in the setting of an experienced team (5). The FET technique was originally intended as single stage procedure for pathologies involving the aortic arch. However, many surgeons have changed their perspective on this. There is ample research evidence of the high rate of subsequent aortic reinterventions regardless of the underlying aortic disease (6–8). Nevertheless, the reintervention rate remains an insufficient parameter for assessing the treatment success of proximal aortic procedures. A composite endpoint for these proximal index procedures is thus needed that also covers morphological and clinical aspects after the procedure such as distal stent graft-induced new entries (dSINE), aortic diameter or aortic-related death that determine distal aortic failure (9, 10).

Aim of this study was to report and to identify possible risk factors for distal aortic failure following the frozen elephant trunk procedure in patients with acute and chronic aortic dissection.

## Patients and Methods

### Ethics Statement

IRB approval was obtained on 04/02/2021 (No. 20-1302) by the institutional review board of the University of Freiburg and the need for informed consent was waived.

### Patients

One hundred eighty-six consecutive patients underwent total aortic arch replacement via the FET technique for acute and chronic aortic dissection at the University Hospital - Heart Centre Freiburg between March 2013 and September 2021. Our cohort was divided into patients with and without distal aortic failure.

### Data Collection and Definition of Parameters

Data was extracted retrospectively from our aortic center’s dedicated database. Acute aortic dissection was defined if symptom onset was fewer than 14 days before hospital admission and chronic thereafter. Stroke was classified according to the VARC-2 criteria using the modified Rankin scale (mRS) and subclassified as disabling stroke (mRS ≥ 3) and non-disabling stroke (mRS ≤ 2) (11). Distal aortic failure was defined as: (I) distal aortic reintervention, (II) aortic diameter dilatation to ≥ 6 cm or growth of > 5 mm within 6 months, (III) occurrence of a dSINE and (IV) aortic-related death. Unknown deaths during follow-up were classified as aortic-related.

### Surgical Technique

Our surgical technique has previously been described in detail (12–15). Briefly, the right axillary artery was routinely used for arterial cannulation. The intended core body temperature was 25 degrees. Bi- (additional selective perfusion cannula placed into the left common carotid artery) or trilateral (additional cannulation of the left axillary artery) antegrade cerebral perfusion was applied depending on the morphology of the Circle of Willis evaluated by preoperative computed tomography angiography (CTA) scans. Bifrontal near-infrared spectroscopy (NIRS) was applied to monitor cerebral oxygenation. Since we routinely implant the 100 mm version of the Thoraflex hybrid-graft (Terumo Aortic, Inchinnan, United Kingdom), cerebrospinal fluid drainage was generally not applied. The stent graft was sized according to the true lumen diameter without oversizing in patients with chronic aortic dissection. Zone 3 anastomoses were performed initially, and since 2017 distal anastomoses have been carried out normally in zone 2. The LSA was anastomosed end-to-end to an 8-mm dacron graft before implantation of the hybrid graft and anastomosed to the FET graft thereafter. This technique facilitates the anastomosis due to limited space at the distal arch. When the end-to-end anastomosis just described is not feasible either for reasons of exposure, because of the poor tissue quality of the native LSA or trilateral cerebral perfusion is done, we use an extra-anatomic approach. In this case, the LSA is closed by a running suture with additional 4.0 Prolene patch–counter–patch sutures or ligature. After that, the LSA prosthetic branch is guided to this location via the second intercostal space, and an end-to-side anastomosis is performed. We applied the beating-heart technique using 300 mL normothermic myocardial perfusion when feasible or cold-blood cardioplegia for myocardial protection (15, 16). We performed a staged approach in patients with chronic aortic dissection already fulfilling the criteria for aortic intervention or replacement in several downstream aortic segments: (I) FET, (II) subsequent thoracic endovascular aortic repair (TEVAR) to the level of the coeliac trunk and (III) open thoraco-abdominal replacement of the remaining involved aortic segments as previously reported (17).

### Aortic Measurements and Follow-Up

CTA was carried out preoperatively, postoperatively, after six months and annually thereafter in at least 3 mm slices using our standard aortic scan protocol. All scans were transferred to imaging software (3mensio, Medical Imaging B.V., Maastricht, The Netherlands) for detailed morphological analysis and measurements of the entire aorta including volume and communication assessment. Follow-up was done at our dedicated aortic outpatient clinic based on our standard follow-up protocol (8).

### Statistical Analysis

IBM SPSS Statistics 27 for Macintosh (Armonk; NY, United States) and R version 3.5.1 (The R Foundation for Statistical Computing, Vienna, Austria) were used for statistical analysis. All values are expressed as number (percentage), mean (standard deviation) or median [interquartile range] depending on normality of the respective values. Normality was assessed graphically using Q-Q plots. Group comparison for the univariable analysis was done via Student’s t-test or Mann-Whitney-U test for continuous and Chi-squared or Fisher’s Exact test for categorial variables when appropriate. A competing risk analysis (competing risk: non-aortic related death) was performed to analyze the influence of clinically selected variables (age, distance from the left subclavian artery to end of dissection, connective tissue disorder, acute aortic dissection, preoperative maximum diameter of the descending aorta and preoperative maximum aorta diameter at the diaphragm level) on the risk for distal aortic failure. Missing values were imputed using predictive mean matching as implemented in the “mice” library (version 3.8.0) of the statistical programming language R. Imputation did not alter any p-values to a noteworthy degree. To compute yearly risk estimates and confidence intervals, we used a cubic smoothing spline. This addresses the scarcity of observations that causes purely non-parametric techniques to suffer from high variance. Smoothing ensures the estimates are more robust and stable.

## Results

### Patients’ Characteristics

Total aortic arch replacement using the FET technique was performed in 186 dissection patients (aged 59 [50-68], 66.1% male). A connective tissue disorder was observed in 12.9%. There were no differences between patients with and without distal aortic failure in terms of demographics and medical history. Eighty-eight patients (47.3%) had already undergone an previous aortic intervention or surgery. Patients’ baseline characteristics are summarized in Table 1.

**TABLE 1:** Demographic data and medical history.

	Total	Distal aortic failure	No distal aortic failure	*p*-value
	*n* = 186	*n* = 88	*n* = 98	
Age (years)	59 [50–68]	60 [48–68]	59 [51–69]	*p* = 0.89
Sex (male)	123 (66.1)	54 (61.4)	69 (70.4)	*p* = 0.22
BMI	26 [23–29]	25 [23–28]	27 [24–29]	*p* = 0.052
BSA (kg/m^2^)	2 [1.8–2.1]	2 [1.8–2.2]	2 [1.9–2.2]	*p* = 0.1
**Cardiovascular risk factors**				
Diabetes (insulin)	5 (2.7)	1 (1.1)	4 (4.1)	*p* = 0.37
Dyslipidaemia	56 (30.1)	27 (30.7)	29 (29.6)	*p* = 0.87
Hypertension	149 (80.1)	72 (81.1)	77 (78.6)	*p* = 0.59
Previous stroke	20 (10.8)	12 (13.6)	8 (8.2)	*p* = 0.25
Previous acute kidney injury	18 (9.7)	7 (8.0)	11 (11.2)	*p* = 0.47
Dialysis	2 (1.1)	0 (0.0)	2 (2.0)	*p* = 0.5
Chronic obstructive pulmonary disease	15 (8.1)	9 (10.2)	6 (6.1)	*p* = 0.42
Coronary artery disease	42 (22.6)	21 (23.9)	21 (21.4)	*p* = 0.73
Connective tissue disorder	24 (12.9)	15 (17.0)	9 (9.2)	*p* = 0.13
**Previous aortic or cardiac surgery**				
Previous surgery	92 (49.5)	44 (50.0)	48 (49.0)	*p* > 0.99
Interval (years)	5 [1–11]	5 [1–10]	5 [1–12]	*p* = 0.52
Coronary artery bypass grafting	7 (3.8)	5 (5.7)	2 (2.0)	*p* = 0.26
Aortic valve replacement	28 (15.1)	18 (20.5)	10 (10.2)	*p* = 0.06
Mitral valve replacement	3 (1.6)	1 (1.1)	2 (2.0)	*p* > 0.99
Ascending replacement	79 (42.5)	39 (44.3)	40 (40.8)	*p* = 0.66
Hemiarch replacement	32 (17.2)	14 (15.9)	18 (18.4)	*p* = 0.7
Others	38 (20.4)	16 (18.2)	22 (22.4)	*p* = 0.59
Aortic Re-do	88 (47.3)	42 (47.7)	46 (46.9)	*p* > 0.99
Re-Sternotomy	76 (40.9)	35 (39.8)	41 (41.8)	*p* = 0.88

*Data are presented as number (%), or median (interquartile range); BMI, body-mass-index; BSA, Body surface area.*

### Aortic Characteristics

Ninety-one (48.9%) patients were treated for acute and 95 (51.1%) for chronic aortic dissection. The most frequent underlying pathology was a residual aortic dissection after previous type A repair (*n* = 68; 36.6%). Significantly more patients were treated for acute type A aortic dissection in the group without distal aortic failure (14.8 vs. 33.7%, *p* = 0.004). The dissection involved the descending thoracic, abdominal aorta and aortic bifurcation in 98.9, 81.4, and 53.1%, respectively. The abdominal aorta was involved more frequently in patients with distal aortic failure (*n* = 77, 91.7% vs. *n* = 67, 72%; *p* < 0.001). There was no difference in the total number of communications between true and false lumen (3.06 ± 2.73 vs. 2.5 ± 2.19, *p* = 0.25) but there were significantly more communications between Ishimaru zone 6-8 (*p* = 0.01) in the distal aortic failure group. The total volume of the thoracic descending aorta measured preoperatively and postoperatively within 36 months after the FET procedure was significantly larger in patients with distal aortic failure. All measured true lumen volumes were similar in both groups, whereas false lumen volume was significant larger preoperatively and within 36 months postoperatively. Detailed aortic characteristics and measurements are provided in the Supplementary Tables 1–2 and Table 2.

**TABLE 2:** Aortic characteristics and measurements (preoperative CTA).

	Total	Distal aortic failure	No distal aortic failure	*p*-value
	*n* = 186	*n* = 88	*n* = 98	
Acute aortic dissection	91 (48.9)	42 (47.7)	49 (50.0)	*p* = 0.77
Type A	46 (24.7)	13 (14.8)	33 (33.7)	*p* = 0.004
Type B	20 (10.8)	12 (13.6)	8 (8.2)	*p* = 0.25
Non-A non-B	25 (13.4)	17 (19.3)	8 (8.2)	*p* = 0.032
Chronic aortic dissection	95 (51.1)	46 (52.3)	49 (50.0)	*p* = 0.77
Residual dissection after previous type A repair	68 (36.6)	31 (35.2)	37 (37.8)	*p* = 0.76
Type B	16 (8.6)	9 (10.2)	7 (7.1)	*p* = 0.6
Non-A non-B	11 (5.9)	6 (6.8)	5 (5.1)	*p* = 0.76
**Diagnostic CTA**				
Dissection extention	*n* = 177	*n* = 84	*n* = 93	
Aortic arch, small curvature	132 (74.6)	57 (67.9)	75 (80.6)	*p* = 0.06
Aotic arch, large curvature	131 (74.0)	57 (67.9)	74 (79.6)	*p* = 0.09
Thoracic descending aorta	175 (98.9)	84 (100.0)	91 (97.8)	*p* = 0.5
Abdominal aorta	144 (81.4)	77 (91.7)	67 (72.0)	*p* < 0.001
Coelic trunk involvement	14 (7.9)	6 (7.1)	8 (8.6)	*p* = 0.78
SMA involvement	28 (15.8)	14 (16.7)	14 (15.1)	*p* > 0.99
Left renal artery involvement	19 (10.7)	7 (8.3)	12 (12.9)	*p* = 0.34
Right renal artery involvement	9 (5.1)	4 (4.8)	5 (5.4)	*p* > 0.99
IMA involvement	1 (0.6)	1 (1.2)	19 (20.4)	*p* = 0.5
Aortic bifurcation	94 (53.1)	52 (61.9)	42 (45.2)	*p* = 0.03
Perfusion abdominal vessels	n = 168	n = 81	n = 87	
CT true lumen	133 (79.2)	61 (75.3)	72 (82.8)	*p* = 0.26
false lumen	11 (6.5)	8 (9.9)	3 (3.4)	*p* = 0.12
Both	24 (14.3)	12 (14.8)	12 (13.8)	*p* > 0.99
SMA true lumen	129 (76.8)	62 (76.5)	67 (77.0)	*p* > 0.99
false lumen	4 (2.4)	3 (3.7)	1 (1.1)	*p* = 0.35
Both	32 (19.0)	16 (19.8)	16 (18.4)	*p* = 0.85
LRA true lumen	92 (54.8)	41 (50.6)	51 (58.6)	*p* = 0.35
false lumen	34 (20.2)	20 (24.7)	14 (16.1)	*p* = 0.18
Both	35 (20.8)	16 (19.8)	19 (21.8)	*p* = 0.85
RRA true lumen	110 (65.5)	51 (63.0)	59 (67.8)	*p* = 0.52
false lumen	23 (13.7)	13 (16.0)	10 (11.5)	*p* = 0.5
Both	29 (17.3)	14 (17.3)	15 (17.2)	*p* > 0.99
Aortic length (mm)	*n* = 177	*n* = 84	*n* = 93	
Anulus to BCT	87 [74-95]	85 [74-96]	88 [74-95]	*p* = 0.73
BCT to LSA	36 [30-42]	35 [30-43]	36 [30-42]	*p* = 0.593
LSA to diaphragm	260 [236-281]	268 [244-290]	256 [228-275]	*p* = 0.006
LSA to CT	281 [258-300]	287 [263-314]	272 [250-294]	*p* = 0.001
LSA to SMA	299 [278-319]	304 [284-334]	289 [269-313]	*p* = 0.002
LSA to LRA	318 [293-339]	320 [307-349]	311 [287-332]	*p* = 0.01
LSA to RRA	314 [291-340]	318 [302-346]	311 [282-329]	*p* = 0.008
LSA to bifurcation	417 [392-446]	420 [395-460]	414 [391-440]	*p* = 0.15
Number of communications	2.76 ± 2.47	3.06 ± 2.73	2.5 ± 2.19	*p* = 0.25
**Number of communications per Ishimaru zone**				
Ishimaru 3	0.14 ± 0.35	0.13 ± 0.34	0.15 ± 0.04	*p* = 0.83
Ishimaru 4-5	1.32 ± 1.58	1.54 ± 1.75	1.12 ± 1.39	*p* = 0.11
Ishimaru 6-8	0.32 ± 0.66	0.44 ± 0.78	0.2 ± 0.5	*p* = 0.01
Ishimaru 9	0.51 ± 0.92	0.58 ± 0.92	0.45 ± 0.92	*p* = 0.21

*Data are presented as number (%) or median (interquartile range); CTA, computed tomography angiography; CT, coeliac trunk; SMA, superior mesenteric artery; LRA, left renal artery; RRA, right renal artery; BCT, brachiocephalic trunk; LSA, left subclavian artery.*

### Intraoperative Data

Concomitant procedures were common. Aortic root replacements (conduits and valve-sparing techniques) were the most frequent ones. The beating-heart technique was applied in 43 (23.1%) patients and trilateral antegrade cerebral perfusion was used in 26 (14.0%) patients. There was no statistically significant intergroup difference between patients with and without distal aortic failure regarding intraoperative data (Table 3).

**TABLE 3:** Intraoperative data and clinical outcomes.

	Total	Distal aortic failure	No distal aortic failure	p-value
Concomitant procedures	*n* = 186	*n* = 88	*n* = 98	
Aortic root conduit	20 (10.8)	10 (11.4)	10 (10.2)	*p* = 0.82
Valve-sparing root replacement	14 (7.5)	4 (4.5)	10 (10.2)	*p* = 0.17
Aortic valve replacement	24 (12.9)	7 (8.0)	17 (17.)	*p* = 0.08
Coronary artery bypass grafting	19 (10.2)	9 (10.2)	10 (10.2)	*p* > 0.99
Operation time (min)	398 [349-471]	398 [337-463]	394 [350-478]	*p* = 0.89
CPB time (min)	213 [175-258]	201 [170-255]	218 [183-262]	*p* = 0.14
Cross-clamp time (min)	122 [95-163]	113 [91-158]	129 [98-164]	*p* = 0.14
Lowest body temperature (°C)	24.8 [24-25.3]	24.7 [24-25.2]	24.8 [24.1-25.3]	*p* = 0.5
Beating-heart technique	43 (23.1)	25 (28.4)	18 (18.4)	*p* = 0.12
Unilateral cerebral perfusion	21 (11.3)	13 (14.8)	8 (8.2)	*p* = 0.25
Bilateral cerebral perfusion	133 (71.5)	65 (73.9)	68 (69.4)	*p* = 0.73
Trilateral cerebral perfusion	26 (14.0)	8 (9.1)	18 (18.4)	*p* = 0.09
Zone 2 distal anastomosis	137 (73.7)	65 (73.9)	72 (73.5)	*p* > 0.99
**Postoperative outcomes**				
In-hospital mortality	15 (8.1)	8 (9.1)	7 (7.1)	*p* = 0.79
Bleeding	20 (10.8)	9 (10.2)	11 (11.2)	*p* > 0.99
Stroke	26 (14.0)	6 (6.8)	20 (20.4)	*p* = 0.011
Disabling stroke	18 (9.7)	4 (4.5)	14 (14.3)	*p* = 0.027
Non-disabling stroke	8 (4.3)	2 (2.3)	6 (6.1)	*p* = 0.28
Dialysis	18 (9.7)	5 (5.7)	13 (13.3)	*p* = 0.13
Paraplegia	4 (2.2)	2 (2.3)	2 (2.0)	*p* > 0.99
Tracheotomy	11 (5.9)	4 (4.5)	7 (7.1)	*p* = 0.55

*Data are presented as number (%) or median (interquartile range), distal aortic failure; CPB, cardiopulmonary bypass.*

### Clinical Outcome

In-hospital mortality was 8.1% while 14% (*n* = 26) suffered a perioperative stroke; 9.7% were classified as disabling strokes and were more frequently observed in patients without distal aortic failure (*p* = 0.011). In-hospital mortality in patients with acute aortic dissection was 12.1% (*n* = 11) and 4.2% (*n* = 4) in patients with chronic aortic dissection. Disabling stroke occurred in 15.4% (*n* = 14, acute aortic dissection) and 4.2% (*n* = 4, chronic aortic dissection), respectively, and Symptomatic spinal cord injury was observed in 4 (2.2%) patients. Postoperative clinical outcomes are summarized in Table 3.

### Distal Aortic Failure and Follow-Up

**FIGURE 1:**
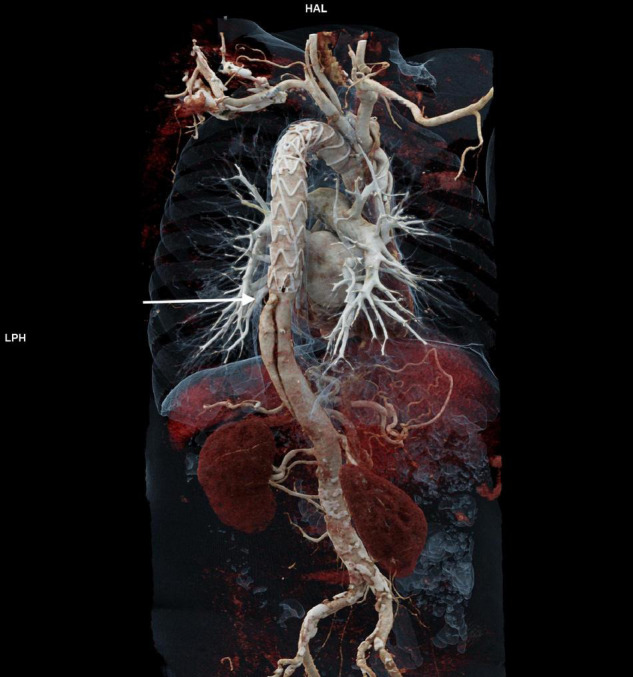
Shows a three dimensional CTA reconstruction of a patient with dSINE (arrow) which causes recurrent false lumen perfusion.

**FIGURE 2:**
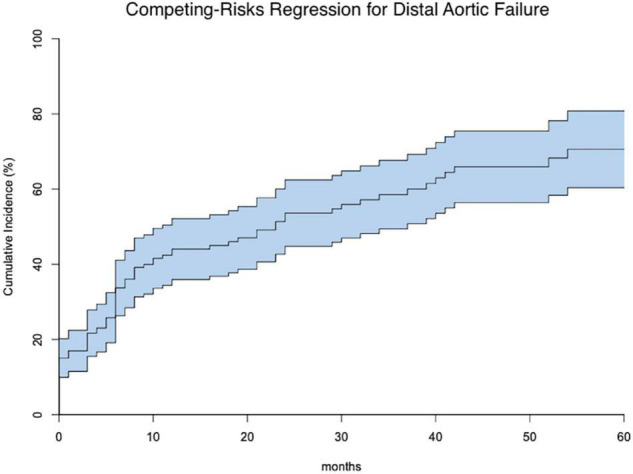
Shows the competing risks for distal aortic failure (middle line) including the 95% confidence intervals within 60 months following the frozen elephant trunk procedure.

One hundred seventy-one patients were discharged. Distal aortic failure occurred in 88 (47.3%) patients: 46 (24.7%) distal reinterventions (see Supplementary Table 3 for indications), 9 (4.8%) aortic diameter dilatations to ≥ 6 cm/ > 5 mm/growth within 6 months, 22 (11.8%) dSINE and 11 (6.4%) aortic-related deaths. Five of the 9 patients with aortic diameter progression and 15 of the 22 dSINE patients underwent an additional aortic reinterventions. Hence, the total number of performed reinterventions cumulates to 66 (35.5%). These reinterventions were done endovascularly in 46 (26.9%), conventionally with open surgery in 7 (4.1%) and via a staged hybrid approach in 13 (7.6%), respectively. The majority of dSINE occurred at the lesser curvature (*n* = 13, 76.5%). Figure 1 shows a three dimensional CTA reconstruction of a patient with dSINE. Median follow-up was complete and 17 (5-4) months. The competitive risk regression model revealed, acute aortic dissection (SHR 2.111; *p* = 0.007), preoperative maximum aortic descending diameter (SHR 1.029; *p* = 0.018) and preoperative maximum aortic diameter at the level of the diaphragm (SHR 1.041; *p* = 0.012) as risk factors for distal aortic failure. The probability of distal aortic failure is as follows: 12 months 26% [95 CI 19-32%], 24 months 42% [95 CI 34-50%], 36 months 51% [95% CI 43-60%], 48 months 60% [95% CI 51-69%] and 60 months 71% [95% CI 60–81%]. The model is shown in Table 4 and illustrated in Figure 2.

**TABLE 4:** Competing risk regression: distal aortic failure.

Variable	*p*-value	SHR	95% CI
Distance left subclavian artery to end of dissection (mm)	0.054	1.002	1.000–1.003
Connective tissue disorder	0.370	1.358	0.628–2.645
Acute aortic dissection,	0.007	2.111	1.224–3.639
Preoperative maximum descending diameter (mm)	0.018	1.029	1.005–1.053
Preoperative maximum diameter diaphragm (mm)	0.012	1.041	1.009–1.075
Age (years)	0.750	0.997	0.979–1.016

## Discussion

The most essential findings of our study can be summarized as: In patients with acute and chronic aortic dissection, (I) total aortic arch replacement via the FET technique is associated with favorable early postoperative outcome; (II) the incidence and risk for distal aortic failure following the FET technique is very high; (III) patients with more acute and more extensive aortic dissections or larger descending aortic diameters in preoperative CTA scans carry a significantly higher risk of developing distal aortic failure.

Our patients’ demographics and medical history are in line with several other reports addressing the issue of total aortic arch replacement (1, 2, 18). In this study, we identified no statistically significant difference in baseline data between patients with and without distal aortic failure. In fact, even an underlying connective tissue disorder was not more common in patients with distal aortic failure and did not prove to be a significant variable in our competing risk regression model, nor was age. Note that this is an important finding, as it reveals that the FET procedure is a durable approach in patients with a connective tissue disorder or of younger age. Therefore, what seems likely is that it is not the disease or age at disease onset *per se* but rather its pathomorphological expression that has the most fundamental impact on treatment durability after total aortic arch replacement via the FET technique.

In this study, significantly more patients without distal aortic failure were initially treated for an acute type A aortic dissection. However, there is a possible bias regarding a shorter follow-up in patients with more adverse events following repair of an acute type A aortic dissection. Correspondingly, postoperative strokes were more frequent in patients without distal aortic failure. As most strokes are of embolic origin, more manipulation might also trigger more embolic events (19). Therefore, both meticulous preoperative evaluation and patient selection are absolutely mandatory. Note that these patients are often already suffering from substantial disability and impairment in daily life, which is why they may tend to avoid making further visits to our outpatient clinic, in turn leading to less follow-up data on patients suffering from potential distal aortic failure. Hence, an even higher distal aortic failure incidence may be possible.

Our study also revealed that the main morphological aspect seems to be aortic enlargement in terms of total downstream aortic volume. More communications between both lumina lead to more false lumen perfusion, which inevitably results in aortic dilatation. Moreover, it seems conclusive that an initially enlarged aorta also carries a higher risk for distal failure (as our regression model shows). Note that although the true lumen appears to have no substantial impact, it is the preoperative and persisting higher false lumen volume postoperatively and during follow-up that plays the main role (12, 20).

Concomitant procedures and intraoperative data were similar between groups and therefore seem to play no major role in the development of distal aortic failure during follow-up. This is conclusive evidence, as no further distal, only proximal procedures, were carried out concomitantly. On the one hand, using the short (100 mm) version of the prosthesis may potentially reduce the risk for symptomatic spinal cord injuries, but it may also raise the risk for distal aortic failure due to less aortic coverage (3). This effect may be aggravated by a trend favoring a zone 2 anastomosis, which simplifies the procedure from the surgical perspective, but proximalis further the distal FET stent-graft landing zone (8).

Distal aortic failure following total arch replacement via the FET technique occurred in about half of our cohort. The high incidence (33%) for aortic reinterventions has been reported before (6), but no risk factor for reinterventions was identified. In our opinion and in the opinion of others (9), reintervention rates *per se* do not sufficiently reflect proximal treatment failure. Distal aortic failure should not be limited to planned or unplanned distal reinterventions but expanded to other distal aortic adverse events during follow up. This necessarily requires a composite endpoint that also includes dSINE, downstream aortic enlargement, and aortic-related death. Taking these factors into account, the probability of distal aortic failure is consequently higher than previously reported reintervention rates. These factors require or might have required additional interventional or surgical treatment to prevent the underlying disease from progressing. Distal aortic failure due to dSINE is a relatively common problem following the FET procedure with higher reported incidences compared to conventional TEVAR (8, 21). One possible explanation may be a more rigid ring at the distal end of the FET stent-graft when using the Thoraflex device (22). Other potential factors are the zone 2 proximalisation of the distal anastomosis that may create a sharp angle of the stentgraft part to the descending’s dissection membrane. Of note, this study also identified an acute aortic dissection as a significant risk factor for developing distal aortic failure following the FET procedure. The dissection membrane is more vulnerable in the acute phase of dissection, whereas it is substantially stiffer and fibrotic in the chronic state. Therefore developing a dSINE is more likely in acute aortic dissections (22).

As this study shows, distal aortic failure can and will occur following the FET procedure. Therefore, we postulate that the designation “single-step approach” is not contemporary anymore for aortic arch replacement via the FET technique. The key variable in determining the long-term success of the FET procedure seems to be the underlying aortic morphology that is crucial to any further decision-making process. This implies two major necessities: (I) a specific follow-up protocol including periodical outpatient visits as well as CTA scans and (II) dedicated aortic teams. Both are mandatory to both detect these events and provide ideal further treatment when needed.

Distal aortic failure often requires invasive treatment in terms of an endovascular extension via TEVAR or open thoracoabdominal replacement. While the Milan group observed no differences in in-hospital mortality comparing both approaches, the overall incidence of adverse events was higher in their open replacement group. They found that respiratory complications were especially likely to have substantial impact (23). We therefore take a three-step approach when treating extensive thoracoabdominal aortic dilatation following the FET procedure. First, we extend the stent-graft part of the FET prosthesis up to the coeliac trunk, usually using two stent-grafts. This approach has so far revealed excellent clinical results with 0% in-hospital mortality or permanent morbidity (8). Applying this strategy, in case of a third-step open surgical repair, we shift the proximal anastomosis more distally and ensure almost continuous ventilation of the left lung, and keep respiratory complications to a minimum during and after the open replacement of the remaining aortic segments (17).

## Limitations

This is a retrospective single-center study with several limitations inherent to this study design. However, this study adds significant new, high quality data on the long-term success and remodeling in patients following the FET procedure.

## Conclusion

In patients with acute and chronic aortic dissection, total aortic arch replacement using the FET technique is associated with very good early postoperative results. However, the incidence and risk for distal aortic failure following the FET procedure is high. In particular, patients with more acute and more extensive aortic dissections or larger descending aortic diameters in preoperative CTA scans carry a substantially higher risk of developing distal aortic failure. The key variable in determining the long-term success of the FET procedure seems to be the underlying aortic morphology that is crucial to any further decision-making process. Hence, the FET technique can no longer be regarded as a single-step therapy for acute and chronic aortic dissection but remains the treatment strategy of first choice for aortic arch and descending aortic pathologies. Moreover, follow-up in dedicated aortic centers is paramount.

## Data Availability Statement

The datasets presented in this article are not readily available because of the requirements of our institutional review board. Individual reasonable requests will be evaluated by the corresponding author. Requests to access the datasets should be directed to TB, tim.berger@uniklinik-freiburg.de.

## Ethics Statement

The studies involving human participants were reviewed and approved by the Institutional Review Board of the University of Freiburg on 04/02/2021 (No. 20-1302). Written informed consent for participation was not required for this study in accordance with the national legislation and the institutional requirements.

## Author Contributions

TB: conceptualization, formal analysis, methodology, and writing—original draft. MG: data curation, formal analysis, and writing—original draft. BR and RG: supervision and writing—review and editing. TW and PD: writing—review and editing. MH: visualization and writing—review and editing. SK: conceptualization, visualization, and writing—review and editing. MC: project administration, supervision, and writing—review and editing. MK: conceptualization, methodology, supervision, validation, and writing—original draft. All authors contributed to the article and approved the submitted version.

## Conflict of Interest

MC and BR are consultants to Terumo Aortic and shareholders of Ascense Medical. MC is consultant to Medtronic, Endospan and NEOS, received speaking honoraria from Cryolife-Jotec and Bentley and is shareholder of TEVAR Ltd. MK has received speaking honoraria from Terumo Aortic. The remaining authors declare that the research was conducted in the absence of any commercial or financial relationships that could be construed as a potential conflict of interest.

## Publisher’s Note

All claims expressed in this article are solely those of the authors and do not necessarily represent those of their affiliated organizations, or those of the publisher, the editors and the reviewers. Any product that may be evaluated in this article, or claim that may be made by its manufacturer, is not guaranteed or endorsed by the publisher.

## Abbreviations

CTA: computed tomography angiography
FET: frozen elephant trunk
LSA: left subclavian artery
SACP: selective antegrade cerebral perfusion.
