# Supplementary material for: Distal Aortic Failure Following the Frozen Elephant Trunk Procedure for Aortic Dissection
Source: Front Cardiovasc Med. 2022 Jun 6;9:911548. doi: 10.3389/fcvm.2022.911548 (PMC9207307; doi:10.3389/fcvm.2022.911548)
Supplement: Supplementary file 1 [file Table_1.DOCX]

| Supplemental Table 1. Volume thoracic aorta (LSA-CT) | | | | |
| --- | --- | --- | --- | --- |
|  | Total | Distal  aortic failure | No distal  aortic failure | p-value |
| Total volume (cm³) |  |  |  |  |
| Preoperative (n=176) | 378 ± 177 | 446 ± 175 | 317 ± 157 | p<0.001 |
| Postoperative (n=138) | 414 ± 184 | 469 ± 183 | 361 ± 169 | p<0.001 |
| 6 Months (n=99) | 417 ± 201 | 476 ± 209 | 336 ± 160 | p<0.001 |
| 12 Months (n=70) | 441 ± 206 | 505 ± 209 | 325 ± 142 | p<0.001 |
| 24 Months (n=48) | 449 ± 217 | 508 ± 228 | 330 ± 132 | p=0.002 |
| 36 Months (n=37) | 469 ± 227 | 530 ± 234 | 356 ± 166 | p=0.011 |
| 48 Months (n=27) | 412 ± 187 | 458 ± 192 | 346 ± 165 | p=0.05 |
| Volume true lumen (cm³) |  |  |  |  |
| Preoperative (n=176) | 128 ± 96 | 140 ± 120 | 117 ± 67 | p=0.83 |
| Postoperative (n=138) | 148 ± 89 | 160 ± 116 | 137 ± 53 | p=0.77 |
| 6 Months (n=99) | 147 ± 85 | 153 ± 100 | 139 ± 60 | p=0.82 |
| 12 Months (n=70) | 155 ± 78 | 159 ± 83 | 146 ± 67 | p=0.7 |
| 24 Months (n=48) | 156 ± 94 | 157 ± 103 | 154 ± 77 | p=0.74 |
| 36 Months (n=37) | 178 ± 97 | 176 ± 104 | 164 ± 84 | p=0.1 |
| 48 Months (n=27) | 157 ± 81 | 150 ± 79 | 166 ± 86 | p=0.61 |
| Volume false lumen(cm³) |  |  |  |  |
| Preoperative (n=176) | 250 ± 159 | 306 ± 156 | 201 ± 147 | p<0.001 |
| Postoperative (n=138) | 266 ± 162 | 309 ± 145 | 225 ± 167 | p<0.001 |
| 6 Months (n=99) | 269 ± 185 | 323 ± 192 | 196 ± 147 | p<0.001 |
| 12 Months (n=70) | 286 ± 202 | 346 ± 213 | 179 ± 127 | p<0.001 |
| 24 Months (n=48) | 293 ± 189 | 351 ± 194 | 176 ± 114 | p<0.001 |
| 36 Months (n=37) | 297 ± 219 | 355 ± 235 | 191 ± 139 | p=0.026 |
| 48 Months (n=27) | 256 ± 178 | 308 ± 183 | 180 ± 146 | p=0.11 |
| Volume true lumen (%) |  |  |  |  |
| Preoperative (n=176) | 36 ± 24 | 31 ± 20 | 41 ± 26 | p=0.03 |
| Postoperative (n=138) | 39 ± 21 | 34 ± 16 | 45 ± 24 | p=0.016 |
| 6 Months (n=99) | 39 ± 21 | 34 ± 18 | 47 ± 23 | p=0.003 |
| 12 Months (n=70) | 40 ± 22 | 35 ± 20 | 49 ± 23 | p=0.004 |
| 24 Months (n=48) | 38 ± 21 | 32 ± 17 | 49 ± 26 | p=0.01 |
| 36 Months (n=37) | 41 ± 24 | 36 ± 23 | 50 ± 25 | p=0.08 |
| 48 Months (n=27) | 43 ± 26 | 36 ± 22 | 53 ± 29 | p=0.162 |
| Volume false lumen (%) |  |  |  |  |
| Preoperative (n=176) | 64 ± 24 | 69 ± 20 | 59 ± 26 | p=0.03 |
| Postoperative (n=138) | 61± 21 | 66 ± 16 | 55 ± 24 | p=0.016 |
| 6 Months (n=99) | 61 ± 21 | 66 ± 18 | 53 ± 23 | p=0.003 |
| 12 Months (n=70) | 60 ± 22 | 65 ± 20 | 51 ± 23 | p=0.004 |
| 24 Months (n=48) | 62 ± 21 | 68 ± 17 | 51 ± 26 | p=0.01 |
| 36 Months (n=37) | 59 ± 24 | 64 ± 23 | 50 ± 25 | p=0.08 |
| 48 Months (n=27) | 57 ± 26 | 64 ± 22 | 47 ± 29 | p=0.162 |

| Supplemental Table 2 Lenght of dissection (mm) | | | | |
| --- | --- | --- | --- | --- |
|  | Total | Distal  aortic failure | No distal  aortic failure | p-value |
| Preoperative (n=162) | 378 ± 147 | 404 ± 120 | 355 ± 165 | p=0.16 |
| Postoperative (n=118) | 335 ± 161 | 368 ± 131 | 306 ± 179 | p=0.19 |
| 6 Months (n=85) | 360 ± 136 | 399 ± 105 | 312 ± 155 | p=0.005 |
| 12 Months (n=66) | 341 ± 166 | 377 ± 148 | 283 ± 181 | p=0.029 |
| 24 Months (n=45) | 367 ±141 | 374 ± 138 | 350 ± 152 | p=0.71 |
| 36 Months (n=33) | 346 ± 162 | 382 ± 141 | 291 ± 182 | p=0.23 |
| 48 Months (n=26) | 367 ± 158 | 407 ± 125 | 312 ± 187 | p=0.28 |
|  |  |  |  |  |

| Supplemental Table 3. Reasons for aortic reintervention | |
| --- | --- |
|  | n=46 |
| Diameterprogression | 33 (71.8) |
| Planned completion | 9 (19.6) |
| Distal true lumen collapse | 1 (2.2) |
| Pseudoaneurysm | 1 (2.2) |
| Impending descending rupture | 1 (2.2) |
| Stent graft dislocation into false lumen | 1 (2.2) |

Data are presented as number (percentage)
